# CnAβ1 shifts cardiac metabolism

**DOI:** 10.18632/aging.101789

**Published:** 2019-01-24

**Authors:** Jesus M. Gomez-Salinero, Pablo García Pavía, Enrique Lara-Pezzi

**Affiliations:** 1 Division of Regenerative Medicine, Ansary Stem Cell Institute, Department of Medicine, Weill Cornell Medicine, New York, NY 10021, USA; 2 Heart Failure and Inherited Cardiac Diseases Unit, Department of Cardiology, Hospital Universitario Puerta de Hierro Majadahonda, Madrid, Spain; 3 Centro de Investigacion Biomedica en Red Cardiovascular (CIBERCV), Madrid, Spain; 4 Facultad de Ciencias de la Salud, Universidad Francisco de Vitoria, UFV, Pozuelo de Alarcón, Madrid, Spain; 5 Centro Nacional de Investigaciones Cardiovasculares (CNIC), Madrid, Spain; 6 National Heart and Lung Institute, Imperial College, London, United Kingdom

**Keywords:** calcineurin, cardiac metabolism, mTOR, Akt, hypertrophy

Cardiovascular diseases have the highest mortality rate worldwide, and their incidence increases with aging. Among them, heart failure after myocardial infarction or maladaptive hypertrophy represents a major health challenge, especially among the elderly. The calcium-regulated phosphatase calcineurin plays a major role in the development of pathological cardiac hypertrophy and heart failure through the activation of the transcription factor NFAT. Calcineurin is composed of a catalytic and a regulatory subunit, CnA and CnB respectively [1]. CnA has 4 major domains: catalytic, CnB-binding, calmodulin-binding and autoinhibitory. In the absence of Ca^2+^, the autoinhibitory domain blocks the catalytic domain, preventing access of the substrate and inhibiting CnA’s activity [2]. Following Ca^2+^ increase in the cytoplasm, the autoinhibitory domain in CnA is removed, the substrate reaches the catalytic domain and is dephosphorylated.

**Figure1:**
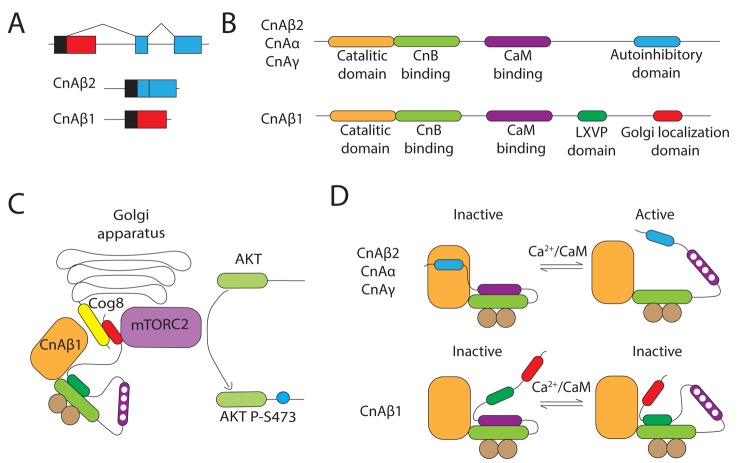
**CnAβ1 alternative signalling promotes activation of the mTORC2/Akt pathway.** (**A**) CnAβ1 is the result of an alternative polyadenylation of Exon 12 in the CnAβ gene. (**B**) CnAβ1’s alternative C-terminal region includes an LXVP motif and a Golgi localization sequence. (**C**) CnAβ1 is localized in the Golgi apparatus through its interaction with Cog8 and modulates mTORC2 phosphorylation of Akt. (**D**) The LXVP inhibitory motif blocks CnAβ1’s catalytic domain even in the presence of Ca2+ and calmodulin. The schematic is based on [3].

Three different genes encode CnA: CnAα and CnAβ are ubiquitously expressed, whereas CnAγ is mainly restricted to brain and testis. Notably, the CnAβ gene expresses an alternative isoform regulated by differential alternative polyadenylation of Exon12 called CnAβ1 (Figure 1A). Contrary to all other CnAs, CnAβ1 lacks the classical autoinhibitory domain and instead contains a unique C-terminal region not present in any other protein. This alternative sequence contains two different α-helixes, comprising an LXVP inhibitory motif and a new Golgi localization signal (Figure 1B) [3,4]. Unlike other calcineurin isoforms, CnAβ1 promotes Akt phosphorylation by mTORC2, rather than NFAT dephosphorylation. Akt activation depends on the localization of CnAβ1 in the Golgi apparatus, which is regulated by its interaction with the Golgi transmembrane protein Cog8 (Figure 1C). The interaction between CnAβ1 and mTORC2 occurs through its alternative C-terminal region and the Golgi localization of CnAβ1 is necessary for the relocalization of mTORC2 from the cytoplasm to the membranes of the cell, and the subsequent phosphorylation of Akt (Figure 1C).

The recent identification of an LXVP motif within the alternative C-terminal region of CnAβ1 has provided a better characterization of its biochemistry [3]. The LXVP peptide was previously found to be a potent inhibitor of CnA activity [2]. The incorporation of an LXVP motif provides CnAβ1’s C-terminal region with a similar function, reducing its phosphatase activity even in the presence of Ca^2+^ and calmodulin (Figure 1D). This is in agreement with previous results showing that a CnAβ1 catalytic-dead mutant had a similar capacity to activate Akt, suggesting that CnAβ1 works as an adaptor protein rather than as a phosphatise [5]. Moreover, the LXVP motif has an unprecedented importance in the context of Ca^2+^ oscillations in the Golgi apparatus.

Unlike all other CnA isoforms, which strongly promote maladaptive cardiac hypertrophy, CnAβ1 reduces hypertrophy by inducing genes involved in the serine and one-carbon metabolic pathway. Activation of this pathway in cardiomyocytes results in reduced protein oxidation in the mitochondria and preserved ATP production, which in turn improves systolic function and prevents adverse ventricular remodelling [6]. Activation of the Akt signalling pathway by CnAβ1 also improves cardiac function after myocardial infarction and promotes skeletal muscle regeneration [5,7,8]. The development of strategies to increase CnAβ1 expression and/or activation of the serine and one-carbon pathway in the heart will increase the quality of patients suffering from maladaptive cardiac hypertrophy or myocardial infarction, and reduce the burden of heart failure, especially among the elderly.
